# Flower-like Silver Bismuth Sulfide/Carbon Nanosphere Nanocomposite for Sensitive Electrochemical Tumor Marker Sensing

**DOI:** 10.1021/acsmeasuresciau.5c00143

**Published:** 2025-11-22

**Authors:** Ragurethinam Shanmugam, Yi-Kuang Yen

**Affiliations:** † Department of Mechanical Engineering, 34877 National Taipei University of Technology, Taipei 10608, Taiwan; ‡ Department of Intelligent Automation Engineering, National Taipei University of Technology, Taipei 10608, Taiwan

**Keywords:** cancer biomarkers, electrochemical biosensor, AgBiS_2_/CNS nanocomposite, screen-printed carbon electrodes (SPCE), aptasensor

## Abstract

A portable electrochemical aptasensor was fabricated using a nanocomposite (Silver Bismuth Sulfide/Carbon Nanosphere, AgBiS_2_/CNS) for the targeted detection of the cancer biomarker carcinoembryonic antigen (CEA). The prepared nanocomposite provides a higher specific surface area, electrical conductivity, and tunable functionality, which enable more effective aptamer immobilization compared to conventional electrode materials. The aptamer, known for its chemical stability and robust surface binding, was immobilized on the surface-treated screen-printed carbon electrode (SPCE), thereby enhancing the reproducibility and stability of the sensor platform. The fabricated portable sensor demonstrated the ability to detect CEA in an early stage with high sensitivity and selectivity. Quantification and optimization were performed using electrochemical impedance spectroscopy (EIS), cyclic voltammetry (CV), and differential pulse voltammetry (DPV) in 0.05 M phosphate buffer solution (PBS, pH 7.0) with 0.1 M KCl [Fe­(CN)_6_]^3–/4–^ (5 mM) electrolyte system. The lower limit of detection (LOD) for CEA was found to be 7.6 ng mL^–1^. Practical applicability was confirmed by evaluating human serum samples, achieving recovery rates in the range of 98.06% to 99.69%.

## Graphical abstract

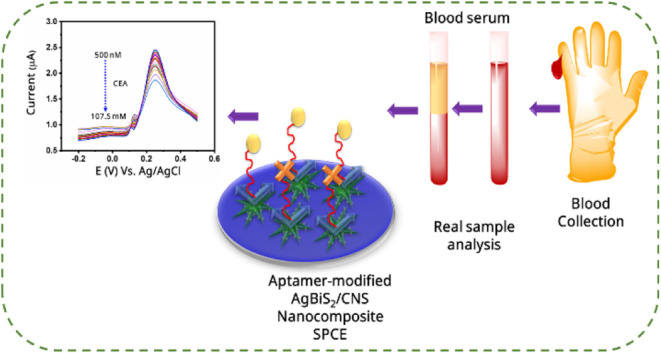

## 1 Introduction

Biomarkers, measurable indicators found in biological substances like blood, urine, or tissue, provide diagnostic insights crucial for improving clinical care of complex illnesses.^1–3^ These biomarkers play a vital role in evaluating and detecting various cancers. As per the World Health Organization (WHO), Cancer is a prominent factor contributing to mortality.^4^ In 2018 and 2020, it accounted for approximately 9.3–9.8 million deaths^5^ and 10 million cancer-related deaths^6^ and is still increasing. Carcinoembryonic Antigen (CEA) is a crucial biomarker and a human glycoprotein that is essential to perform and control several metabolic functions that include cancer morbidity and mortality. In addition, it aids effective functions in controlling the rapid growth of metastatic cancer cells.^7^ The elevated levels of CEA play a vital role in diagnosing and identifying several malignant cancers and diseases earlier. In this regard, the identification of exceptionally sensitive and selective analytical scrutinization methods plays a vital role in the earlier diagnosis of several cancers.^8^ When normal cells turn malignant, serum CEA levels rise quickly; in healthy persons, the typical range is less than 5.0 ng mL^–1^.^9,10^ Fluorescent immunoassay,^11^ enzyme-linked immunosorbent assay (ELISA),^12^ Radioimmunoassay (RIA),^13^ Immunoradiometric assay (IRMA),^14^ and colorimetric methods are the several methods available for the detection of CEA. Even though these methods tend to possess several merits, they have disadvantages such as the need for skilled professionals, low sensitivity, accuracy, and complex procedures.^15^ Electrochemical methods provide several advantages over other techniques, including low cost, simple design, and portability. These platforms are capable of performing sensitive and selective detections, even in complex biological fluids like serum.^16^ Biosensor technology has evolved to address limitations of conventional analytical methods by providing high sensitivity, selectivity, rapid response, accuracy, and simple procedures through the use of biorecognition elements.^17^ In potentiometric devices, analytical information is generally obtained by converting a biological response or biochemical reaction into a potential signal. This can be achieved through a variety of transducers, including ion-selective electrodes (ISEs), field-effect transistors (FETs), and enzyme-based potentiometric sensors, among others.^18^ The transducer converts the specific interaction of biorecognition molecules (such as aptamers) with target biomarkers into measurable electrical signals. In our study, we employed electrochemical transducers, where the aptamer’s stable three-dimensional structure enhances target binding by expanding the contact region between the aptamer and the biomarker.^19^ Compared to antibodies, aptamers are simpler, have better chemical stability, are less expensive, easier to immobilize, and have higher selectivity.^20^ Therefore, Grabowska et al. created a technique that uses SPE-PEI/rGO (Screen-printed electrodes-polyethylenimine/reduced graphene oxide)-aptamer-based electrochemical sensors for brain natriuretic peptide (BNP-32) and cardiac troponin I (cTnI).^21^ Naseri et al. suggested using a novel multivalent aptamer immobilized on the surface of the screen-printed gold electrode (aptamer/SPGE) to develop the first electrochemical aptasensor for label-free detection of lactoferrin with a wide dynamic detection range.^22^ GE/TDN–aptamer/MCH (Gold electrode/tetrahedral DNA nanostructures-aptamer/6-mercapto-1-hexanol) is a highly sensitive and selective electrochemical dual-aptamer biosensor for specific detection of biomarker (Human Epidermal Growth Factor Receptor 2) HER2 by Ou et al.^23^ Among the various nanomaterials employed for the electrochemical detection of biomarkers, silver bismuth sulfide (AgBiS_2_) has shown promising characteristics. AgBiS_2_ is composed of earth-abundant, nontoxic elements and can be synthesized *via* low-cost and scalable methods such as hydrothermal, solvothermal, sonochemical, microwave, and polyol routes.^24–32^ Beyond its ease of preparation, AgBiS_2_ exhibits favorable charge transfer kinetics, good conductivity, chemical stability, and high surface activity, all of which are advantageous for electrochemical sensing. Although a structural phase transition from hexagonal β-AgBiS_2_ to cubic α-AgBiS_2_ occurs at ∼468 K.^26,27^ Among these methods, the hydrothermal method possesses several advantages, such as an easy procedure, homogeneous preparation, and morphology. In addition, the focus lies on its room-temperature electrochemical properties that promote sensitive biomarker detection.

Carbon nanospheres (CNSs) have also attracted significant interest due to their high surface area, porous structure, and good electrical conductivity. Their stable physical and chemical properties, biocompatibility, and ability to provide abundant anchoring sites make them highly suitable for immobilizing biomolecules, including aptamers.^33–36^ While CNSs have been widely explored in catalysis, energy storage, and drug delivery, their porous conductive network can also enhance electron transport and increase loading capacity in electrochemical biosensors.^33^ Additionally, because ultrafine carbon nanospheres—which are typically less than 200 nm—are readily internalized into cells by intracellular endocytosis, they have been successfully used in pharmaceutical and biological applications, including the administration of medications, genes, proteins, and imaging agents.^34,35^ Since carbon nanospheres have stable physical and chemical properties, a large specific surface area, an abundant porous structure, outstanding electrical conductivity, and biocompatibility, they are currently a popular choice for adsorbents, drug carriers, and hydrogen storage materials. Carbon nanosphere adsorbents have been utilized extensively in wastewater treatment to adsorb organic contaminants, heavy metal ions, and colors in aqueous solutions.^36^ In this work, we combine AgBiS_2_ with CNSs to form a synergistic nanocomposite. CNSs provide a stable, conductive, and high-surface-area scaffold for aptamer immobilization, while AgBiS_2_ contributes to high redox activity and charge transfer properties. Together, this hybrid structure enhances both sensitivity and selectivity, making it well-suited for the electrochemical detection of CEA, an important tumor biomarker. The nanocomposite AgBiS_2_/CNS was prepared by utilizing the precursor and employing a hydrothermal-assisted ultrasonication method. Furthermore, the nanocomposite was modified with biorecognition molecules called aptamers that aid in the targeted detection of CEA. In addition, the synthesized nanocomposite was scrutinized through several physicochemical methods. It has been implemented in the sensing of CEA through CV, DPV, and EIS techniques. Further, the fabricated portable sensor was employed in the practical applicability analysis of the biological and environmental samples.

## 2 Experimental Section

### 2.1 Chemicals and Reagents

Silver nitrate (AgNO_3_, ACS reagent, ≥99.0%), Bismuth­(III) nitrate pentahydrate (Bi­(NO_3_)_3_·5H_2_O, ACS reagent, ≥98.0%), Ethylene glycol (HOCH_2_CH_2_OH, ≥99.0%), Polyethylene glycol (H­(OCH_2_CH_2_)_*n*_OH), Thiourea (NH_2_CSNH_2_, ACS reagent, ≥99.0%) and all other chemicals were procured from Sigma-Aldrich, Taiwan. Sodium dihydrogen phosphate monohydrate (NaH_2_PO_4_·H_2_O, ACS reagent, ≥98.00%) and sodium phosphate dibasic (Na_2_HPO_4_, ACS reagent, ≥99.00%), potassium hexacyanoferrate (III) (K_3_Fe (CN)_6_ (s), ACS reagent, ≥99.00%), potassium hexacyanoferrate (II) trihydrate (K_4_Fe­(CN)_6_·3H_2_O, ACS reagent, ≥98.50%), potassium chloride (KCl (s), ACS reagent, ≥98.50%), sodium hydroxide (NaOH, reagent grade, ≥98%, pellets (anhydrous)), and ethanol (CH_3_CH_2_OH, ACS reagent, 95.0%) were also obtained from Sigma-Aldrich, Taiwan. The aptamer sequence (5′-NH_2_-GTGACGCTCCTAACGCTGACTCAGGTGCAGTTCTCGACTCGGTCTTGATGTGGGTCCTGTCCGTCCGAACCAATC-3′) was synthesized and purified by All Bio Science Inc., Taiwan. The electrolyte (0.05 M PBS) was prepared by mixing NaH_2_PO_4_·H_2_O and Na_2_HPO_4_ with the aid of distilled water (DI).

### 2.2 Instruments

Powder X-ray diffraction (XRD) was utilized for the crystallographic scrutinization of the prepared nanomaterials with the aid of the XPERT-PRO spectrometer (PANalytical B.V., The Netherlands) with Cu-Ka radiation λ = 1.54056 Å. X-ray photoelectron spectroscopy (XPS) was performed by a Thermo ESCALAB 250 instrument. Field emission scanning electron microscope (FE-SEM) images and their composition were investigated by using energy-dispersive X-ray (EDX) spectroscopy (FE-SEM, Quanta 250, F.E.G., Hitachi, Japan, operated at 15 kV). The electrochemical experiments were performed on a PalmSens4 electrochemical workstation equipped with PS Trace 5.9 software (PalmSens BV, Houten, The Netherlands). Three electrode systems were utilized for the detection of cancer biomarkers, including screen-printed carbon electrodes (SPCE) as the working electrode, Silver/Silver chloride (Ag/AgCl) as the reference electrode, and platinum wire as the counter electrode.

### 2.3 Preparation of AgBiS_2_/CNS Nanocomposite

In 60 mL of ethylene glycol (EG), 0.01 mol of AgNO_3_ and 0.01 mol of Bi­(NO_3_)_3_·5H_2_O were combined and agitated until completely dissolved. The aforementioned solution was first given 0.1 mmol of polyethylene glycol (PEG) as a soft template, and it was then allowed to rest at 40 °C for 4 h before receiving 0.01 mol of thiourea. The finished solution was homogenized by stirring it, then put into a 100 mL Teflon-lined stainless-steel autoclave and heated to 180 °C for 20 h. It was then allowed to spontaneously cool to room temperature. Using distilled water and ethanol, the synthesized product was obtained by centrifuging multiple times. The finished black product was vacuum-dried at 60 °C for 8 h. Further, the nanocomposite was prepared by adding an equal ratio of CNS to the prepared AgBiS_2_ and ultrasonicated for 2 h.^37^ The external force provided by the ultrasonication method generates the local shear stresses that are ultimately responsible for dispersion.^38^ It helps in the homogeneous dispersion of the CNS on the surface of AgBiS_2_. Then the procured AgBiS_2_/CNS nanocomposite was dried and collected (Scheme 1).

**Scheme 1:**
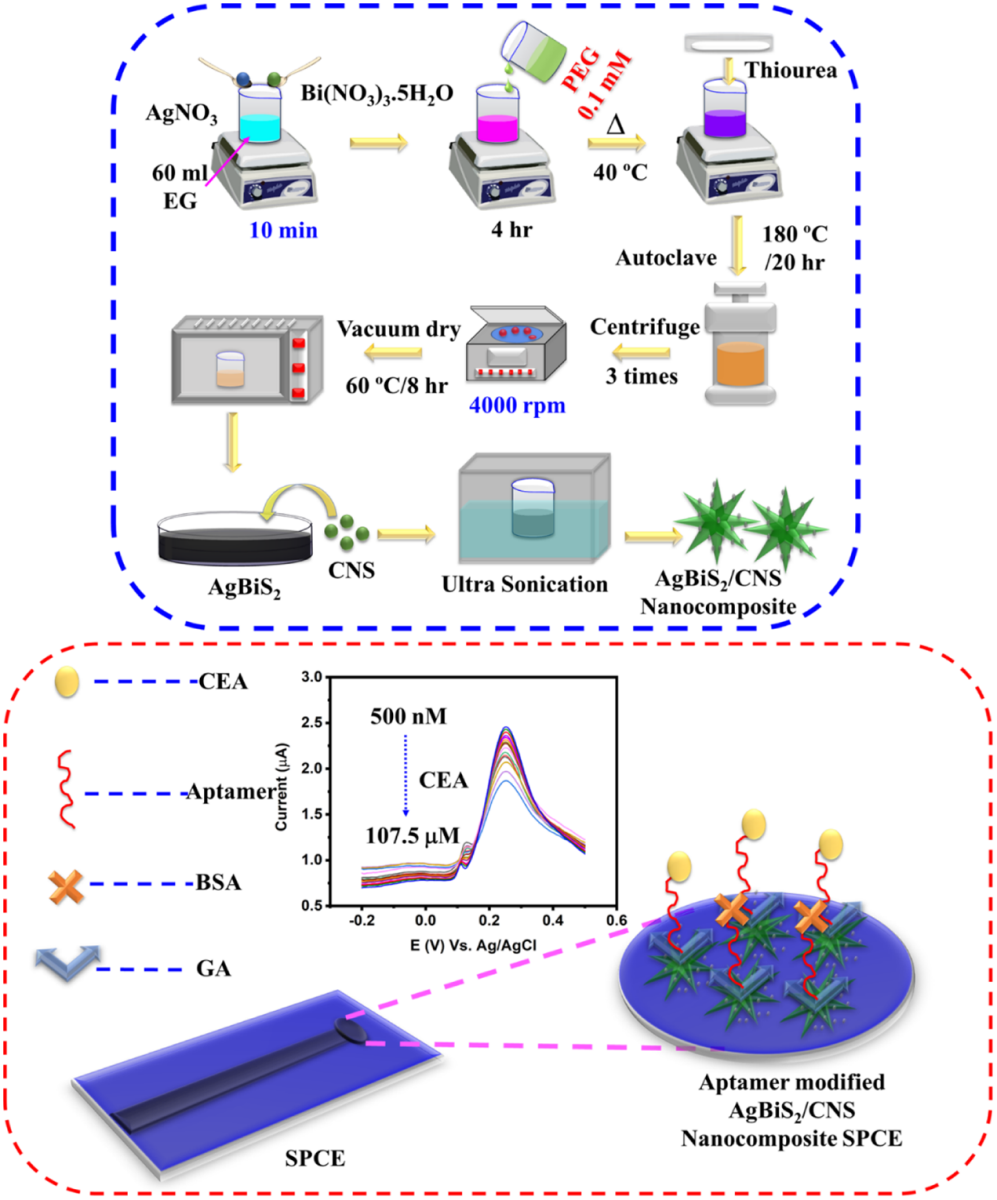
Schematic Illustration of the Preparation of AgBiS_2_/CNS Nanocomposite (Left)^a^DVCB^a^

a Electrochemical detection of various cancer biomarkers through the fabricated aptamer-modified AgBiS_2_/CNS nanocomposite screen-printed carbon electrode (Right, Inset: DPV current response for CEA). CEA – Carcinogenic embryonic antigen; BSA – Bovine serum albumin; GA – Glutaraldehyde; SPCE – Screen-Printed Carbon Electrode.

### 2.4 Design and Fabrication of Cancer Biosensor

The screen-printed carbon electrode (SPCE) was employed as the substrate for the biosensor. First, the prepared AgBiS_2_/CNS nanocomposite was dispersed in DI water to make a suspension. 8 μL of the suspension was then drop-cast on the surface of a SPCE. The prepared AgBiS_2_/CNS nanocomposite was then modified with an aminylated aptamer through glutaraldehyde (GA). First, GA was dropped on the surface of the nanocomposite, and then the aminylated aptamer was added to the biosensor.^39^ Bovine serum albumin (BSA) was introduced into the sensor system, which binds and blocks the unoccupied electrode surfaces and remaining active sites of the electrode. The total assay time for each sample to be prepared was around 20 h. Finally, the fabricated aptamer-modified AgBiS_2_/CNS nanocomposite biosensor was implemented in the detection of CEA biomarkers (Scheme 1).

### 2.5 Real Sample Preparation

The blood serum samples were purchased from Sigma-Aldrich, Taiwan. To prepare blood serum samples, 1.0 mL of blood serum is diluted in 50 mL of distilled water. The solution is then centrifuged at 6000 rpm for three intervals of 30 min each. After centrifugation, the supernatant is collected for further processing. The real sample analysis was performed using the prepared blood serum with the aid of a fabricated aptamer-modified AgBiS_2_/CNS nanocomposite SPCE.

## 3 Results and Discussion

### 3.1 Structural and Morphological Characterizations

We prepared AgBiS_2_ nanomaterials through the simple hydrothermal method, and the AgBiS_2_/CNS nanocomposite was prepared by the ultrasonication method. The obtained nanocomposite was successfully examined through various characterization techniques (Figure 1A). First, the prepared materials were scrutinized through powder XRD, which exemplifies the corresponding hkl planes with **JCPDS no: 89-3672**, thereby confirming the successful formation of the nanocomposite. Precisely, the AgBiS_2_ pattern tends to exhibit hkl planes, which correspond to the previous literature; 27.32°, 31.52°, 34.37°, 45.27°, and 52.62° correspond to (111), (200), (220), (311), and (222). For CNS, the XRD pattern exemplifies (002) and (100) planes for peaks at 24.12° and 43.60°. The average particle size of the synthesized nanomaterials has been calculated with the aid of Scherrer’s equation (eq 1).^40^

1 D=Kλ/βcos⁡θ

**Figure 1:**
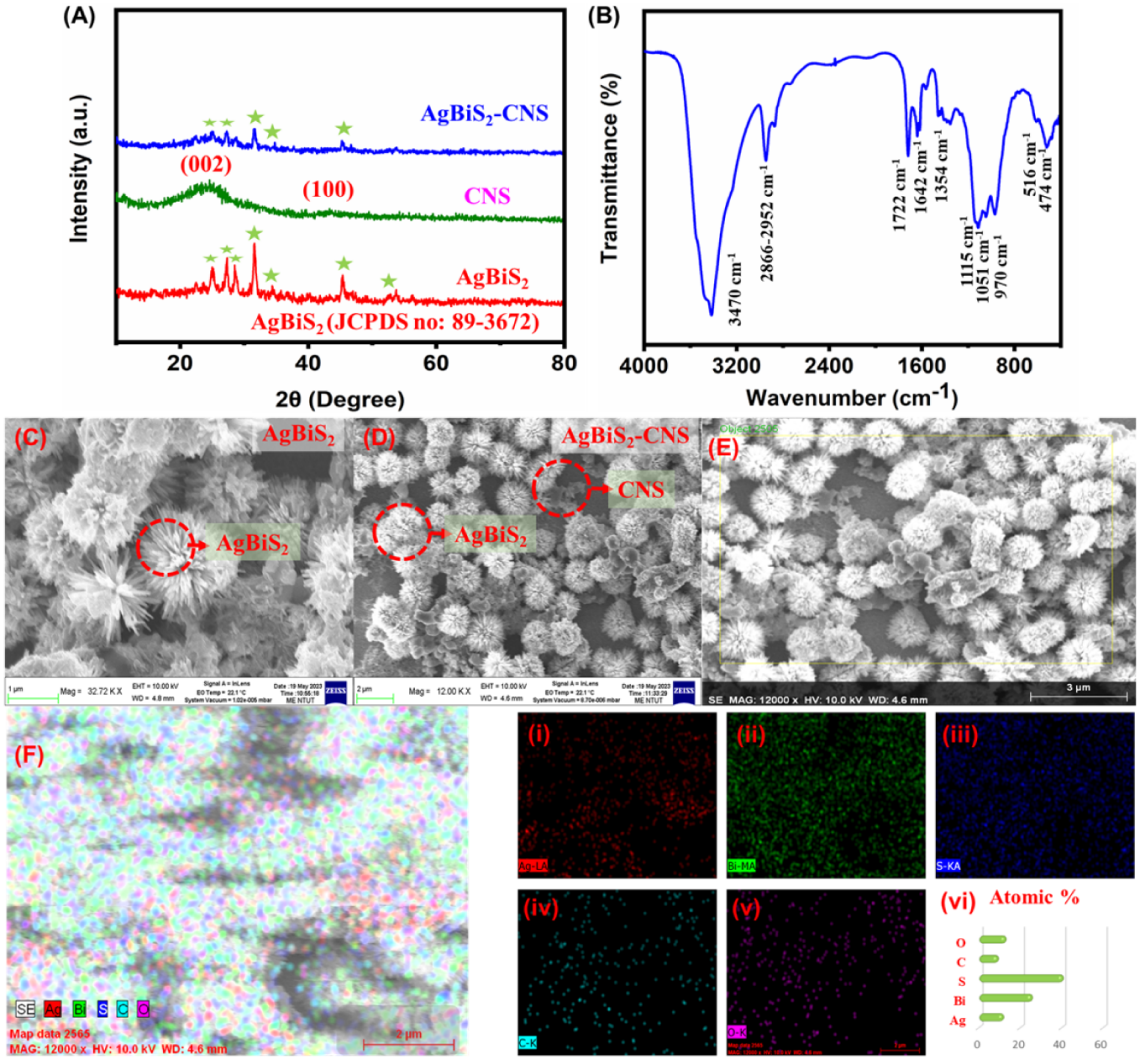
**(A)** XRD pattern of AgBiS_2_, CNS, and AgBiS_2_/CNS nanocomposite, **(B)** FTIR spectra for the aptamer-modified AgBiS_2_/CNS nanocomposite, **(C)** SEM images of AgBiS_2_, **(D–E)** AgBiS_2_/CNS nanocomposite, (F) Elemental mapping of AgBiS_2_/CNS nanocomposite **(i)** Ag, **(ii)** Bi, **(iii)** S, **(iv)** C, **(v)** O, and **(vi)** Bar graph for various elements on the nanocomposite.

The Scherrer constant, X-ray wavelength, Bragg angle, and diffraction angle of the Full Width at Half Maximum (FWHM) in radians are represented by the letters *K*, λ, β, and θ in eq 1. The average particle size was found to be 256 nm, 49 nm and 153 nm for the AgBiS_2_, CNS, and AgBiS_2_/CNS nanomaterials.

Further, the nanocomposite was morphologically examined through a SEM. AgBiS_2_ exemplifies nanoflower-like morphology as illustrated in the image (Figure 1C), and Figure 1D exhibits a nanosphere morphology for CNS. From the FESEM analysis, elemental mapping was performed. The respective results are illustrated in Figure 1E. Figure 1F exhibits the mixed elemental mapping of the AgBiS_2_/CNS nanocomposite with the homogeneous distribution of all the elements present in the composite (Ag, Bi, S, C, O) in Figure 1F (i–v). Furthermore, the elemental composition (Figure 1F (vi) and Figure S1) was scrutinized, and the weight percentages were calculated to be 11.74% (Ag), 25.63% (Bi), 40.54% (S), 9.18% (C), and 12.71% (O). From the obtained results, the homogeneous distribution and presence of all the respective elements in the nanocomposite were scrutinized.

Further, the functional groups of the aptamer-modified AgBiS_2_/CNS nanocomposite were successfully characterized through Fourier transform infrared (FTIR) spectroscopy in Figure 1B. The functional bands procured at 474 cm^–1^ and 516 cm^–1^ attributed to the AgBiS_2_ lattice vibrations.^41^ The vibrational bands observed at 3470 cm^–1^ and 1724 cm^–1^ were attributed to the surface-absorbed water and hydroxyl groups.^42,43^ The peaks at 1354–1642 cm^–1^ correspond to the −CONH and from 2866–2952 cm^–1^ attributed to the −CH group of BSA proteins.^44^ The peaks at 1722 cm^–1^ related to the aldehyde group (C=O) of GA and the peaks procured at 970 cm^–1^, 1051 cm^–1^, and 1115 cm^–1^ correlated to the deoxyribose skeleton correspond to C–C/C–O, P–O, and C–O stretch.^45–47^ These peaks confirm the presence of DNA aptamers on the nanocomposite surface. From the obtained results, the successful formation and modification of the aptamer-modified AgBiS_2_/CNS nanocomposite were scrutinized. Further, it has been utilized for electrochemical aptasensing of CEA.

### 3.2 XPS Spectroscopy Characterizations

The chemical composition and valence state of the AgBiS_2_/CNS nanocomposite were analyzed using XPS, and the detailed data are presented in Figure 2A–F. The comprehensive survey spectrum of AgBiS_2_/CNS nanocomposite revealed prominent characteristic peaks at 377.7, 162.6, 162.37, 288.3, and 536.6 eV, which correspond to Silver (Ag 3d), Bismuth (Bi 4f), Sulfur (S 2p), oxygen (O 1s), and carbon (C 1s), respectively. Figure 2B presents the high-resolution core-level spectrum of Ag 3d, showing prominent peaks at 367.5, 372.1, and 377.8 eV, corresponding to Ag 3d_5/2_ and Ag 3d_3/2_, respectively.^48^ These values are consistent with reported data, indicating that the silver is in the Ag^1+^ state. Additionally, strong peaks observed at 162.3 and 163.62 eV correspond to Bi 4f_7/2_, while peaks at 167.6 and 168.9 eV are associated with Bi 4f_5/2_ (Figure 2C). These deconvoluted peaks indicate the presence of Bi and Bi–S species, corroborated by the XRD peaks of AgBiS_2_, with the secondary phase attributed to excess sulfur in the material.^49^ The core-level XPS spectrum of S 2p (Figure 2D) shows significant peaks at 162.3, 166, and 168 eV, corresponding to the S 2p peaks of pristine AgBiS_2_ samples.^50,51^ Furthermore, the XPS spectrum of C 1s (Figure 2E) displays prominent peaks at 283.6, 286.77, 288.3, and 292.5 eV, associated with sp^2^ hybridized carbon, hydroxyl, and epoxy groups, respectively, consistent with aptamer immobilization.^52^ Figure 2F shows the core-level spectrum of O 1s, with a significant peak at 536.6 eV and deconvolution revealing three main peaks at 532.7, 534.1, and 536.5 eV, corresponding to lattice oxygen, adsorbed oxygen, and chemisorbed oxygen molecules.^53^ These peaks are consistent with surface functionalization. From the obtained results, it is evident that the AgBiS_2_/CNS nanocomposite was successfully prepared with the significant distribution of the elements. Therefore, it can be effectively employed in the electrochemical aptasensing of CEA.

**Figure 2:**
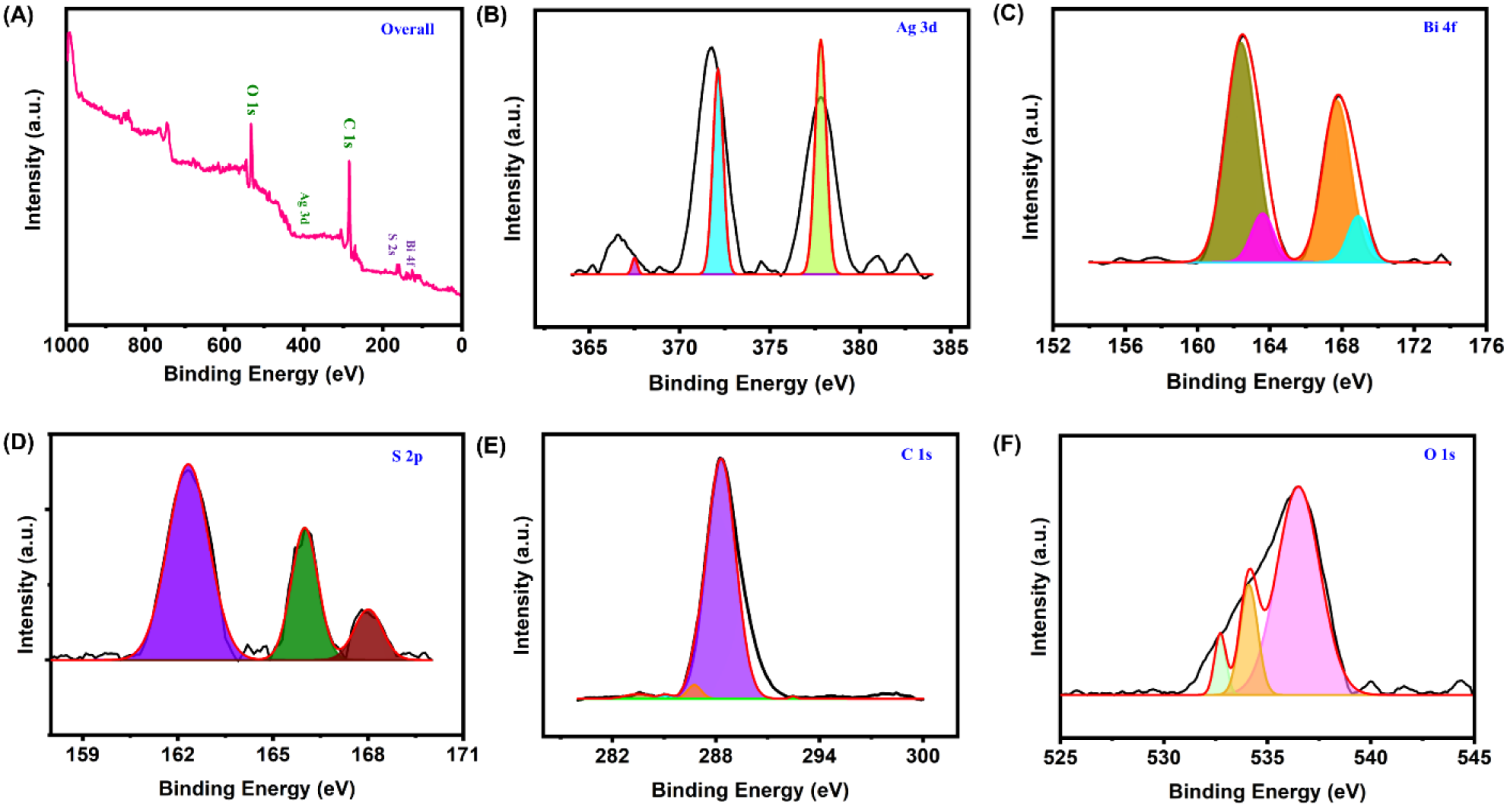
XPS survey of AgBiS_2_/CNS nanocomposite: **(A)** Overall, **(B)** Ag 3d, **(C)** Bi 4f, **(D)** S 2p, **(E)** C 1s, and **(F)** O 1s.

### 3.3 Electrochemical Characterizations and Optimizations

EIS proves to be a valuable instrument for examining the electrochemical characteristics of electrodes subjected to various modifications. This technique holds significance in the realm of electrochemistry, as it involves measuring impedance in a circuit using ohms as the unit of resistance. Essentially, EIS employs an alternating current (AC) approach wherein a modulated potential is applied over time at a consistent frequency with a small amplitude (approximately 5 mV). The investigation conducted through EIS sheds light on the electron transfer kinetics occurring between the electrode and electrolyte within the electrochemical system. Figure 3A displays the impedance spectra through the Argand plot. The semicircle portion observed at higher frequencies is indicative of the charge transfer resistance (*R*_ct_), while the low-frequency region corresponds to Warburg diffusion (*Z*_w_). Additionally, the double-layer capacitance is represented by *C*_dl_, and the solution resistance is denoted as *R*_s_. EIS spectra for bare SPCE, AgBiS_2_, CNS, AgBiS_2_–CNS, AgBiS_2_–CNS/GA, and AgBiS_2_–CNS/GA/Aptamer-modified electrodes were performed in the 0.1 M KCl [Fe­(CN)_6_]^3–/4–^ (5 mM) system over a frequency range of 100 mHz to 100 kHz at an amplitude of 5 mV. Therefore, Figure 3A reveals the R_ct_ capabilities of the bare SPCE and other modified electrodes. In particular, compared to the composite-modified electrodes, the GA-modified electrodes exhibit an enhanced kinetic performance. From the obtained plots, the CNS SPCE and AgBiS_2_–CNS/GA-modified SPCE tend to exhibit reduced charge transfer resistance and increased electron transfer ability compared to the other electrodes. The aptamer-modified SPCE shows comparatively reduced *R*_ct_ due to the insulating nature of the aptamer layer, which partially blocks electron transfer. This confirms that while aptamer immobilization is essential for selective CEA recognition, it slightly reduces electron transfer efficiency compared to GA or composite-modified electrodes. The detailed analysis and the Nyquist plot parameters are listed in Table 1.

**Figure 3:**
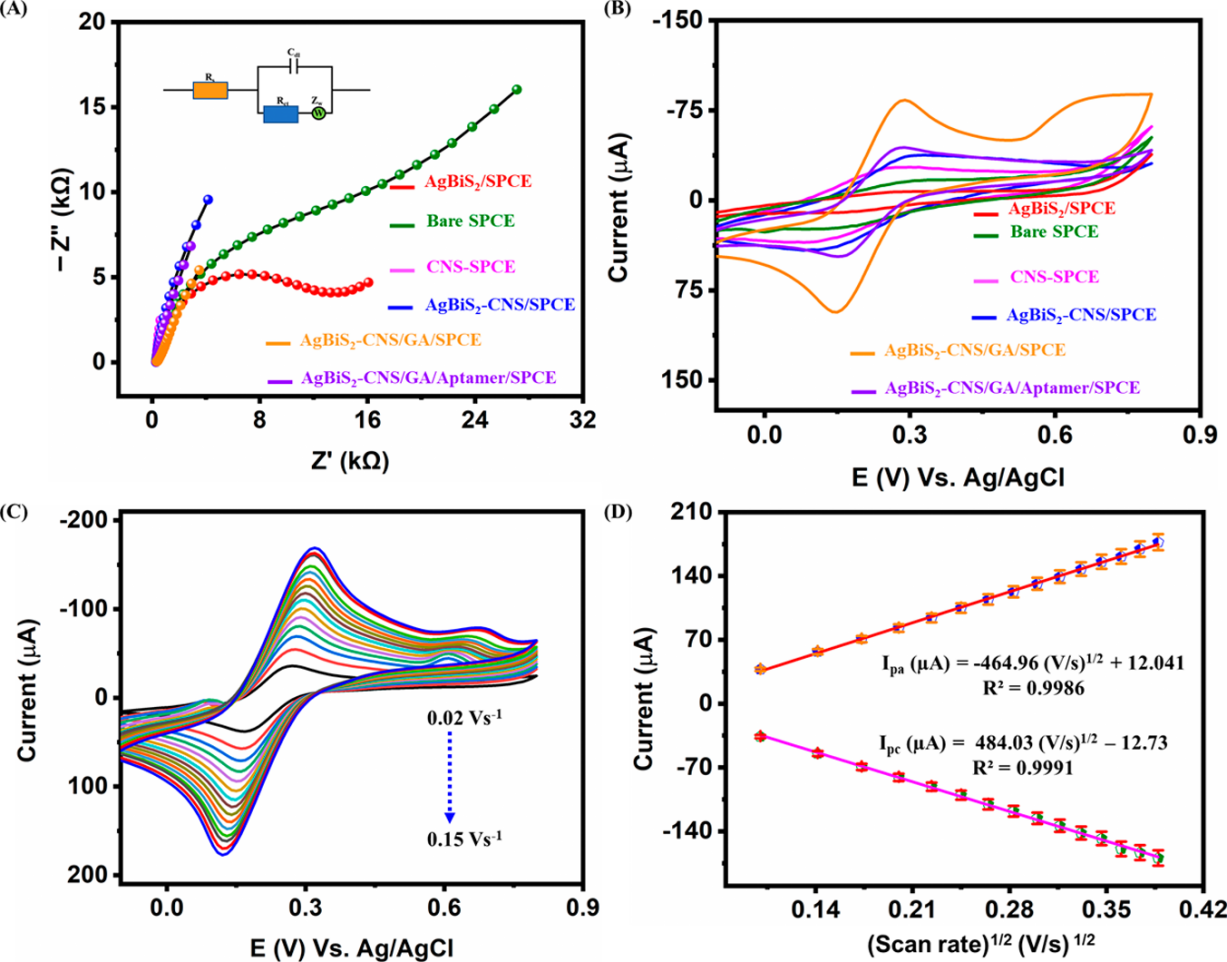
**(A)** Nyquist plot for the modified electrodes that include bare SPCE, AgBiS_2_, CNS, AgBiS_2_–CNS, AgBiS_2_–CNS/GA, and AgBiS_2_–CNS/GA/Aptamer modified electrodes, **(B)** Cyclic voltammetry curves for all the modified electrodes, **(C)** Varying scan rate (0.02–0.15 V s^–1^), and **(D)** Corresponding calibration plots for the varying scan rate *vs.* peak currents. All of the measurements were performed in the 0.1 M KCl [Fe­(CN)_6_]^3–/4–^ (5 mM) system.

**Table 1:** Nyquist Plots and the Acquired Parameters of the Bare SPCE and the Modified Electrodes

Electrode	*R*_ct_ (kΩ)	*A* (cm^2^)	*I*_pa_ (μA)	*I*_pc_ (μA)	*E*_pa_ (V)	*E*_pc_ (V)
**Bare SPCE**	11.890	0.031	23.77	–16.57	0.119	0.319
**AgBiS**_**2**_ **SPCE**	12.368	0.049	10.13	–7.03	0.149	0.259
**CNS SPCE**	0.310	0.062	34.54	–27.48	0.099	0.269
**AgBiS**_**2**_ **–CNS SPCE**	0.080	0.162	41.03	–37.40	0.099	0.309
**AgBiS**_**2**_ **–CNS/GA SPCE**	0.0416	1.125	93.05	–83.66	0.146	0.149
**AgBiS**_**2**_ **–CNS/GA/Aptamer SPCE**	0.069	0.173	46.80	–43.94	0.289	0.280

In comparison, the AgBiS_2_–CNS/GA/aptamer-modified SPCE electrode tends to show elevated charge transfer resistance due to the binding of the aptamer with the modified electrode surface. This, in turn, increases the amount of loading on the surface of the electrode, leading to increased resistance. Further, the cyclic voltammetry (CV, Figure 3B) analysis was performed with all the modified electrodes in the presence of a 0.1 M KCl [Fe­(CN)_6_]^3–/4–^ (5 mM) system at a potential window of −0.1 to +0.8 V. From the obtained results, bare SPCE displays a reduced peak current due to the lack of electron transfer ability. Up next, the AgBiS_2_-modified SPCE shows comparatively increased resistance with the reduced peak current compared to the bare SPCE. In addition, the CNS-modified SPCE exemplifies improved conductivity with reduced charge transfer resistance due to its extensive characteristics. Among the various electrodes, the AgBiS_2_–CNS/GA-modified SPCE electrode tends to express an elevated current response due to the increased electrotransfer capabilities and the reduced charge transfer resistance. In the case of AgBiS_2_–CNS/GA/Aptamer modified SPCE, the current response tends to be comparatively decreased due to the bulk accumulation on the modified electrodes due to the binding of the aptamer. Further, the CV analysis through varying scan rates (0.02–0.15 V s^–1^) in the presence of a 0.1 M KCl [Fe­(CN)_6_]^3–/4–^ (5 mM) system with the aid of AgBiS_2_–CNS/GA/Aptamer modified SPCE as illustrated in Figure 3C. The corresponding calibration plot between the square root of the scan rate versus peak currents is depicted in Figure 3D. The procured CV results unveil that the response demonstrates exceptional linearity with the linear regression equations of *I*_pa_ = 484.03 (V/s)^1/2^–12.73 and *I*_pc_ = −464.96 (V/s)^1/2^ + 12.04 and regression coefficient (*R* ^2^) of 0.9991 and 0.9986. From the results, the electrochemically active surface area (EASA) has been evaluated through the Randles–Sevick equation (eq 2), and the values obtained from Figure 3D and Figure S2.^54^

2 $$I_{p}=2.69\times 10^{5}n^{3/2}AD^{1/2}C\gamma^{1/2}$$

*I*_p_ – the peak anodic or cathodic current (A),

*n* – the number of electron transfers (n),

*A* – the electroactive area (cm^2^),

D – the diffusion coefficient of [Fe­(CN)_6_]^3–/4–^ (cm^2^ s^–1^),

γ – the scan rate (V s^–1^),

*C* – the concentration of the [Fe­(CN)_6_]^3–/4–^ (M cm^–3^) solution.

On evaluation, the procured EASA values are elaborated in Table 1. Among the various electrodes, the AgBiS_2_–CNS/GA/Aptamer-modified SPCE exhibits an EASA of 0.173 cm^2^, which is our primary electrode for the detection of carcinoembryonic Antigen (CEA).

### 3.4 Detection of CEA through AgBiS_2_-–CNS/GA/Aptamer-Modified SPCE

CV was implemented in the sensing of CEA through AgBiS_2_–CNS/GA/Aptamer-modified SPCE in the presence of 0.05 M PBS (pH 7.0) with 0.1 M KCl [Fe­(CN)_6_]^3–/4–^ (5 mM) system. Foremost, the concentration of CEA was varied from 50 to 300 μM at a scan rate of 0.05 V s^–1^, as depicted in Figure 4A. From the obtained results, the fabricated electrodes exhibit a good linear current response upon varying the concentration linearly. The current response tends to decrease linearly due to the higher level of binding of the aptamer with the CEA. The developed sensor operates through a surface-blocking mechanism rather than a structure-switching aptamer. Specifically, aptamers are immobilized on the AgBiS_2_/CNS nanocomposite-modified SPCE *via* glutaraldehyde coupling. The immobilized aptamer layer is relatively insulating, which partially hinders the electron transfer at the electrode interface. Upon binding with the target CEA biomarker, this steric hindrance further increases, resulting in a reduced current response in the voltammetric signal. Thus, the decrease in current is attributed to the blocking effect of aptamer immobilization and subsequent aptamer–CEA interactions, which restrict electron transfer. Further, the linearity has been confirmed through the procured linear regression equation (Figure 4B) of *I*_pa_ (μA) = −0.0067 (μM) + 3.2761 and *I*_pc_ (μA) = 0.0066 (μM) – 3.1853 and regression coefficients (*R* ^2^) of 0.9997 and 0.9974. Up next, the scan rate analysis was performed by varying the scan rate from 0.01 to 0.1 V s^–1^ with the aid of AgBiS_2_–CNS/GA/Aptamer-modified SPCE in the presence of 0.05 M PBS (pH 7.0) with 0.1 M KCl [Fe­(CN)_6_]^3–/4–^ (5 mM) system (Figure 4C). Upon increasing the scan rate the respective current tends to increase linearly due to the polarization effect. In addition, this linearity has been evident through the procured linear regression equation (Figure 4D) between scan rate versus peak currents with *I*_pa_ (μA) = 26.842 (V s^–1^) + 0.3032 and *I*_pc_ (μA) = 0.0066 (μM) – 3.1853 and *R* ^2^ of 0.9997 and 0.9974. From the results, the optimized scan rate was further utilized for the detection of CEA using AgBiS_2_–CNS/GA/Aptamer-modified SPCE. The pH analysis was performed with the aid of AgBiS_2_–CNS/GA/Aptamer-modified SPCE in the presence of 0.05 M PBS (pH 7.0) with 0.1 M KCl [Fe­(CN)_6_]^3–/4–^ (5 mM) system, as shown in Figure 4E. Among the various pH values, pH 7.0 tends to show an elevated current response; therefore, pH 7.0 has been utilized for further sensing analysis. Figure 4E exhibits the calibration plots between varying pH versus peak current and peak potential with a linear regression equation of *E*_pa_ (*V*) = −0.0112 pH + 0.387 and *R* ^2^ = 0.9584.

**Figure 4:**
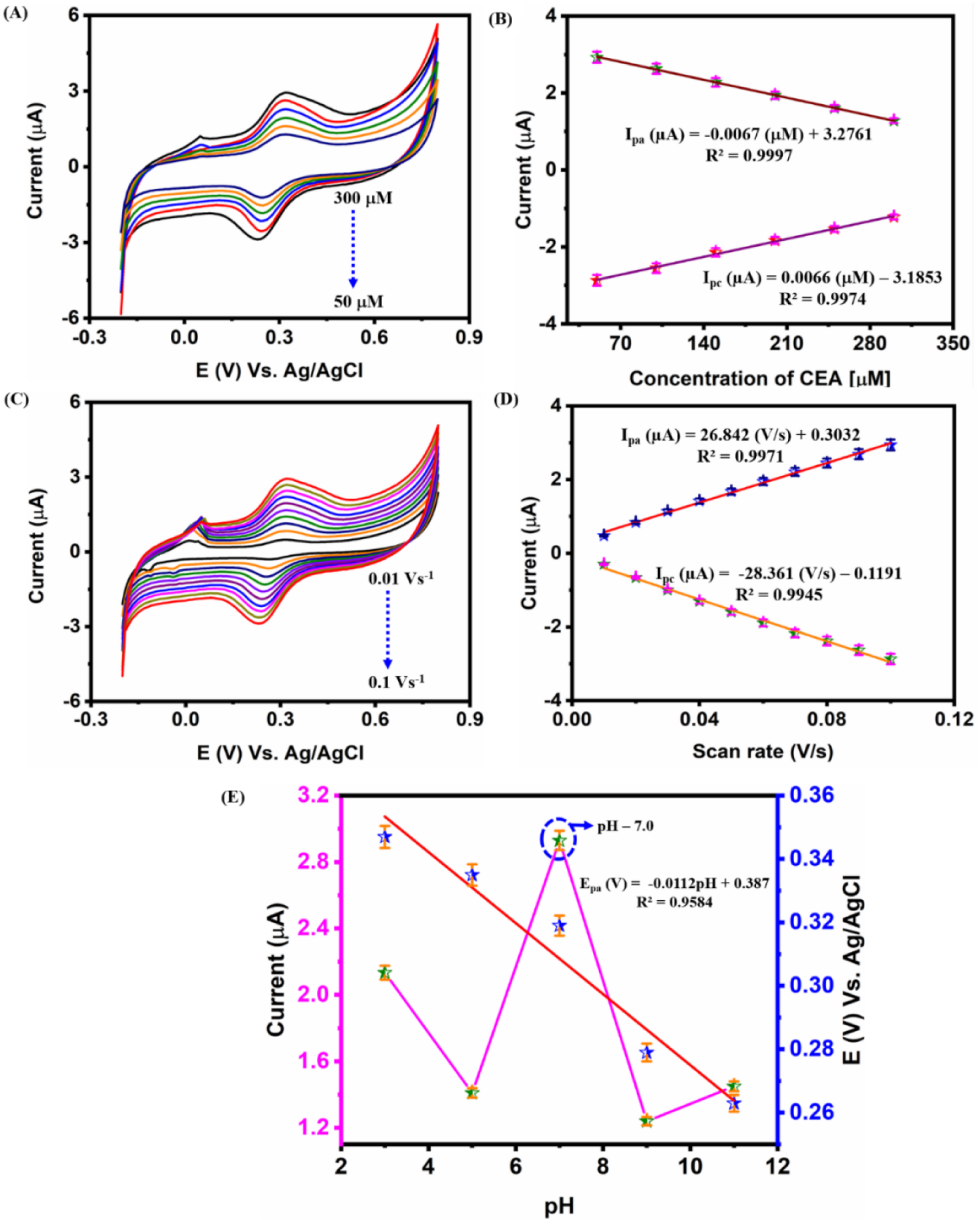
**(A)** Cyclic voltammetry (CV) response for varying addition of CEA (50–300 μM) at a scan rate of 0.05 V s^–1^, **(B)** Corresponding calibration plot for varying concentration of CEA versus peak anodic and cathodic current, **(C)** CV current of varying scan rates (0.02–0.1 V s^–1^), **(D)** Calibration plots for varying scan rates versus peak cathodic and anodic currents, and **(E)** Calibration plots for varying pH versus peak current and peak potential. All measurements were performed in the presence of 0.05 M PBS (pH 7.0) with a 0.1 M KCl [Fe­(CN)_6_]^3–/4–^ (5 mM) system.

### 3.5 Differential Pulse Voltammetry (DPV) Analysis for the Detection of CEA

Differential Pulse Voltammetry (DPV), known for its heightened sensitivity, was employed to detect CEA using AgBiS_2_–CNS/GA/Aptamer-modified SPCE. This detection was carried out in the presence of 0.05 M PBS (pH 7.0) with 0.1 M KCl [Fe­(CN)_6_]^3–/4–^ (5 mM) system, as illustrated in Figure 5A. The current response was analyzed by varying the concentration from 500 nM to 107.5 μM. When the concentration of CEA increases, the current response tends to decrease due to the higher binding of the aptamer with CEA, thereby increasing the deposition on the electrode surface and causing an increase in resistance. The linear regression equation (Figure 5B and Figure S3) was procured to be *I*_pa_ (μA) = −0.0048 (μM) + 2.414 and a regression coefficient (*R* ^2^) of 0.9805. The limit of detection (LOD), limit of quantification (LOQ), and sensitivity were analyzed using eqs 3 and 4 as follows:^55^

3 $$LOD=3S_{a}/b$$

4 $$LOQ=10S_{a}/b$$

**Figure 5:**
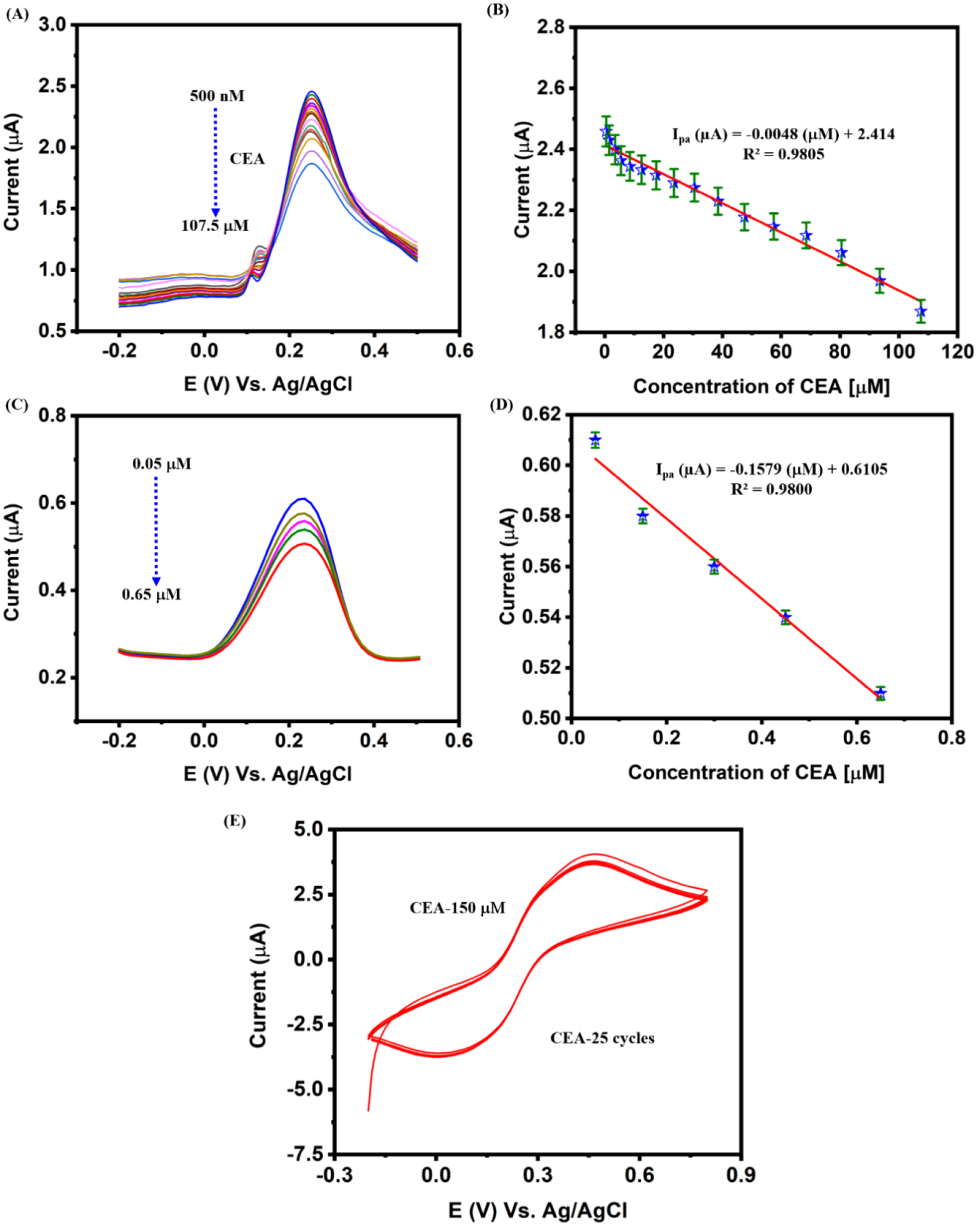
**(A)** Differential pulse voltammetry (DPV) response for varying addition of CEA (500 nM–107.5 μM), **(B)** Corresponding calibration plot for varying concentration of CEA versus peak current, **(C)** DPV current response for the real sample analysis by varying addition of CEA with the aid of blood serum, **(D)** Calibration plots for blood serum versus peak current, and **(E)** Storage stability analysis for modified SPCE toward CEA for 25 cycles. All measurements were performed in the presence of 0.05 M PBS (pH 7.0) with a 0.1 M KCl [Fe­(CN)_6_]^3–/4–^ (5 mM) system.

“*S*_a_” – the standard deviation of ten blank responses,

“*b*” – the slope acquired from the linear calibration plot.

Upon evaluation, the LOD, LOQ, and sensitivity were found to be 7.6 ng mL^–1^, 22.8 ng mL^–1^, and 0.0245 μA μM^–1^ cm^–2^. Moreover, the obtained results exemplify a lower detection limit with appraisable sensitivity.

### 3.6 Comparison Studies

In comparative studies, previously reported materials and methods for the detection of CEA were evaluated against our proposed sensor, as shown in Table 2. Among these, the photoelectrochemical immunoassay (PEC) stands out for its commendable accuracy, broad specificity, and high sensitivity. Other methods have been noted for their specific advantages but also have limitations, such as restricted temperature ranges, short shelf lives, expensive equipment, and the need for skilled personnel. In contrast, our electrochemical sensor combines AgBiS_2_ nanoparticles with CNS to provide a high surface area, enhanced conductivity, and efficient electron transfer, while glutaraldehyde cross-linking ensures stable aptamer immobilization. This material combination results in improved signal amplification, lower LOD, rapid response, and high stability compared with previously reported electrochemical sensors. These features make the proposed sensor highly suitable for point-of-care applications, demonstrating superior practical applicability for CEA detection in biological samples.

**Table 2:** Comparative Performance of the AgBiS_2_–CNS/GA/Aptamer-Modified SPCE for the Detection of CEA

Sensor fabrication	Detection method	Linear range	Detection limit	Reference
^a^Anti-CEA/WO_3_@BiOI@CdS/ITO	PEC	0.01–50 ng mL^–1^	3.2 pg mL^–1^	^56^
^b^ZnO flower-rods modified with g-C_3_N_4_–Au nanoparticle nanohybrids	PEC	0.01–2.5 ng mL^–1^	1.9 pg mL^–1^	^57^
^c^Au nanoparticle decorated graphene modified glassy carbon electrode	EC	0.1–1000 ng mL^–1^	60 pg mL^–1^	^58^
^d^Anti-CEA/AuNP@nafion/FC@CHIT/GCE	EC	0.01–150 ng mL^–1^	0.003 ng mL^–1^	^59^
Anti-CEA/nafion/FC@CHIT/GCE	EC	0.03–100 ng mL^–1^	0.03 ng mL^–1^	^59^
AgBiS_2_–CNS/GA/Aptamer-modified SPCE	DPV	90–19350 ng mL^–1^	7.6 ng mL^–1^	**This work**

a Anti-CEA/WO_3_@BiOI@CdS/ITO – Antibody-carcinoembryonic antigen/Tungstic anhydride@Bismuth oxyiodide@cadmium sulfide/indium-tin oxide.

b ZnO flower-rod/gC_3_N_4_ – Zinc oxide/graphitic carbon nitride.

c Au nanoparticle – Gold nanoparticles.

d CEA/AuNP@nafion/FC@CHIT/GCE – Antibody carcinoembryonic antigen/Gold nanoparticles@nafion/redox species K_3_Fe­(CN)_6_@chitosan/glassy carbon electrodes.

### 3.7 Real Sample Analysis for the Detection of CEA

Real sample analysis was performed in the presence of CEA through AgBiS_2_–CNS/GA/Aptamer-modified SPCE by varying the amount of CEA (0.05–0.65 μM). The working parameters of real sample analysis were performed with the aid of DPV in the potential range of −0.2 to 0.5 V. The standard addition procedure was followed for the evaluation of blood serum. The known concentration of CEA was spiked by the standard addition method into the diluted solution. When the blood serum was added to the electrolyte, it tended to produce a corresponding current response (Figure 5C). Upon the linear addition of the blood serum into the system, the respective linear current responses were obtained, as illustrated in Figure 5D. The linear regression equation was procured to be *I*_pa_ (μA) = −0.1579 (μM) + 0.6105 and *R* ^2^ of 0.9800. Further, the recovery results of the procured current responses were plotted in Table S1. From the results, the recovery percentage was found to be in the range of 98.06–99.69% with a relative standard deviation (RSD) in the range of 0.63–3.69%. Therefore, the fabricated portable AgBiS_2_–CNS/GA/Aptamer-modified SPCE demonstrates a good practical applicability performance.

### 3.8 Stability, Selectivity, Repeatability, and Reproducibility for the Detection of CEA through AgBiS_2_–CNS/GA/Aptamer-Modified SPCE

The storage stability of the AgBiS_2_–CNS/GA/Aptamer-modified SPCE portable sensor was evaluated through the detection of CEA (150 μM) for 25 continuous cycles (Figure 5E). Upon evaluation, almost 96% of its initial current was retrieved with an RSD of 2.56%. Therefore, it can be a promising candidate for the fabrication of portable sensors for the detection of CEA. The selectivity of the fabricated sensors was evaluated by introducing similar cancer biomarkers, biological drugs, biomolecules, and ions in the presence of 0.05 M PBS (pH 7.0) with a 0.1 M KCl [Fe­(CN)_6_]^3–/4–^ (5 mM) electrolyte system (Figure 6A). First and foremost, the cancer biomarker CEA (150 μM) was introduced in the electrochemical environment followed by the introduction of various interferents that include NSE (1 × Neuron Specific Enolase, ∼150 μM), GLU (2 × Glucose, ∼300 μM), DA (2 × Dopamine, ∼300 μM), AA (2 × Ascorbic Acid, ∼300 μM), UA (2 × Uric Acid, ∼300 μM), CHL (2 × Cholesterol, ∼300 μM), and 5 times excess addition of Ca^2+^, Na^+^, and K^+^ (∼300 μM). Upon analysis, the current response was procured to be 99.84% (NSE), 99.67% (GLU), 99.23% (DA), 99.04% (AA), 98.91% (UA), 98.79% (CHL), 98.64% (Ca^2+^), 98.51% (Na^+^), and 98.22% (K^+^) for the various interferents respectively (Figure 6B). The selectivity assay was conducted by introducing CEA into the electrochemical system, followed by various potential interferents (NSE, glucose, dopamine, ascorbic acid, uric acid, cholesterol, Ca^2+^, Na^+^, and K^+^). Although the sensor exhibited strong anti-interference performance, this assessment is limited by the absence of control tests without CEA and the lack of structurally similar tumor biomarkers such as AFP or PSA. Future work will involve performing these additional control and cross-reactivity studies to provide a more comprehensive evaluation of selectivity. From the selectivity analysis, the fabricated sensor tends to possess a high selectivity with very low interference. In addition, the repeatability of the sensors was scrutinized by evaluating the performance of the portable sensors for five measurements (Figure 6C and D). Upon analysis, the sensor tends to exhibit good repeatable performance with 99.03% of the initial current response for the detection of CEA. Moreover, the reproducibility of the fabricated portable sensor was scrutinized by measuring the response in 5 different modified electrodes. From the obtained results, it was stated clearly that 99.15% of the initial current responses were rectified while utilizing five different modified electrodes (Figure 6E and F). In addition, the storage stability analysis for 25 days reveals ∼93% of its initial current response, as shown in Figure S4. From the above-obtained results, it is evident that the fabricated portable AgBiS_2_–CNS/GA/Aptamer-modified SPCE sensor can be an influential candidate for the detection of CEA biomarkers with enhanced performance with lower detection limit, sensitivity, selectivity, reproducibility, and repeatability.

**Figure 6:**
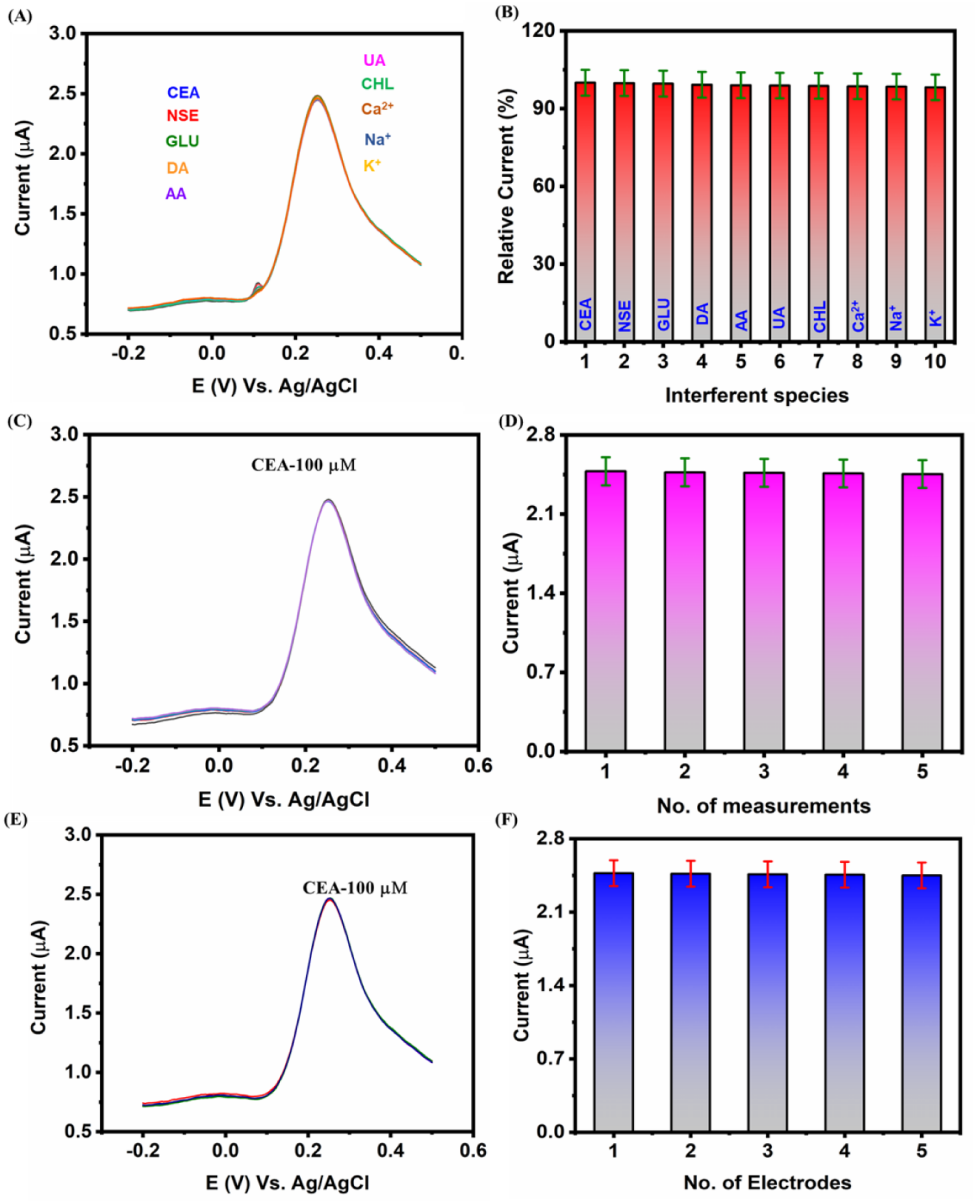
**(A)** DPV response for the selectivity analysis of CEA versus various biological interferents (CEA, NSE, GLU, DA, AA, UA, CHL, Ca^2+^, Na^+^ and K^+^), **(B)** Corresponding calibration plot for various interferents versus relative current (%), **(C)** DPV current response for the repeatability analysis for the detection of CEA, **(D)** Histogram analysis for repeatable measurements versus peak current, and **(E)** DPV current response for the reproducibility analysis using 5 different electrodes for the detection of CEA, **(F)** Histogram analysis for reproducibility analysis using 5 different electrodes versus peak current. All measurements were performed in the presence of 0.05 M PBS (pH 7.0) with a 0.1 M KCl [Fe­(CN)_6_]^3–/4–^ (5 mM) system.

## 4 Conclusion

In conclusion, we successfully developed a portable electrochemical sensor using an aptamer-modified AgBiS_2_/CNS nanocomposite. The nanocomposite was synthesized through a hydrothermal-assisted ultrasonication method and characterized using various physicochemical techniques. The aptamer was immobilized on the nanocomposite-altered electrode with GA, while BSA was introduced to prevent unwanted binding on the aptamers. The fabricated electrode, applied for the detection of the cancer biomarker CEA, demonstrated exceptional electrochemical performance, including a low detection limit (7.6 ng mL^–1^), sensitivity, selectivity, repeatability, reproducibility, and storage stability. It also offers a higher active surface area, electron transfer ability, and specific surface area. The practical feasibility analysis of the fabricated sensor exhibits recovery results of 98.06–99.69% in the human serum sample. Overall, the aptamer-modified AgBiS_2_/CNS nanocomposite sensor shows great promise for detecting CEA in diverse biological and environmental samples, offering reliable results in real sample analysis.

## Supplementary Material



## Notes

Information on EDX elemental composition, EIS analysis, the conversion formula, recovery analysis, and stability has been provided (PDF)
