# Supplementary material for: Flower-like Silver Bismuth Sulfide/Carbon Nanosphere Nanocomposite for Sensitive Electrochemical Tumor Marker Sensing
Source: ACS Meas Sci Au. 2025 Nov 22;6(1):158–69. doi: 10.1021/acsmeasuresciau.5c00143 (PMC12921616; doi:10.1021/acsmeasuresciau.5c00143)
Supplement: Supplementary file 1 [file tg5c00143_si_001.pdf]

Supporting Information

Flower-Like Silver Bismuth Sulfide/Carbon Nanosphere Nanocomposite for Sensitive Electrochemical Tumor Marker Sensing

Ragurethinam Shanmugam [a] *and Yi-Kuang Yen* [a, b*]

[a] Department of Mechanical Engineering, National Taipei University of Technology, Taipei, 106, Taiwan

[b] Department of Intelligent Automation Engineering, National Taipei University of Technology, Taipei, 106, Taiwan

[*]**Corresponding author:** ykyen@ntut.edu.tw

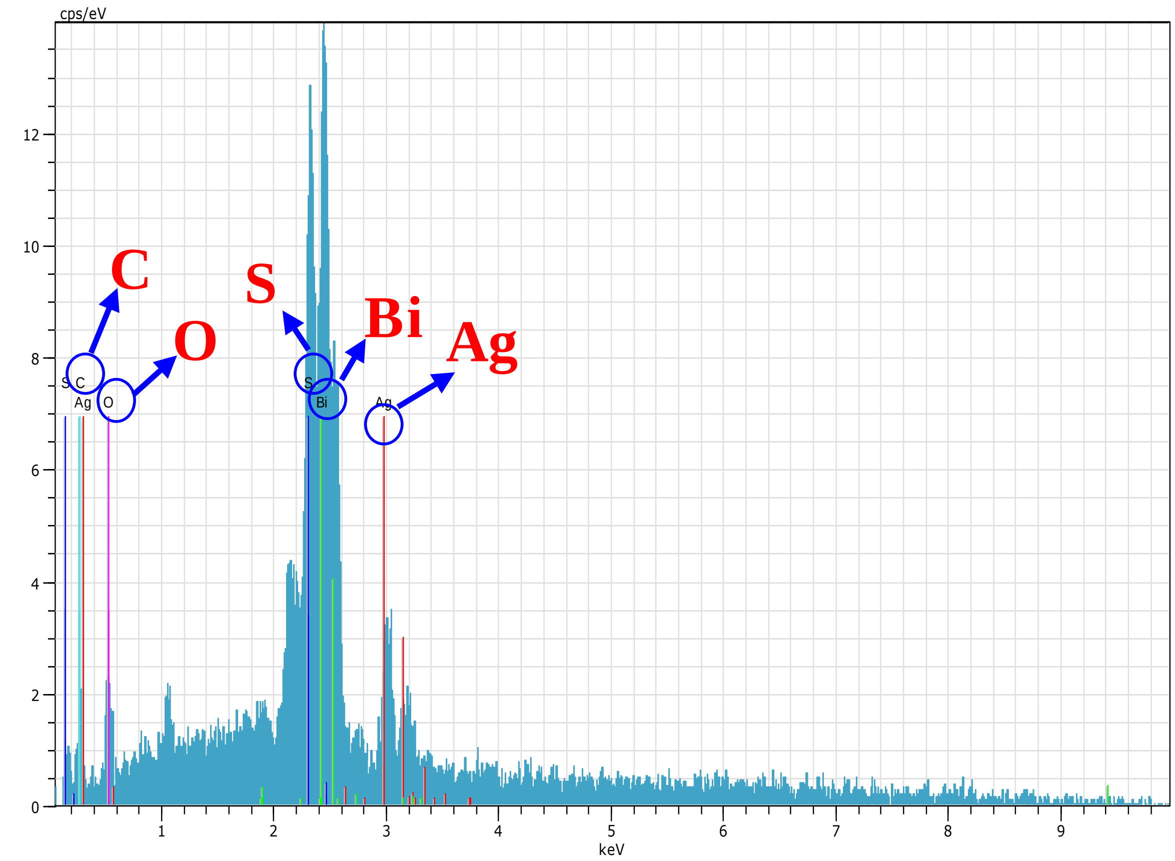

Fig. S1. EDX elemental composition of $AgBiS_2$/CNS nanocomposite.

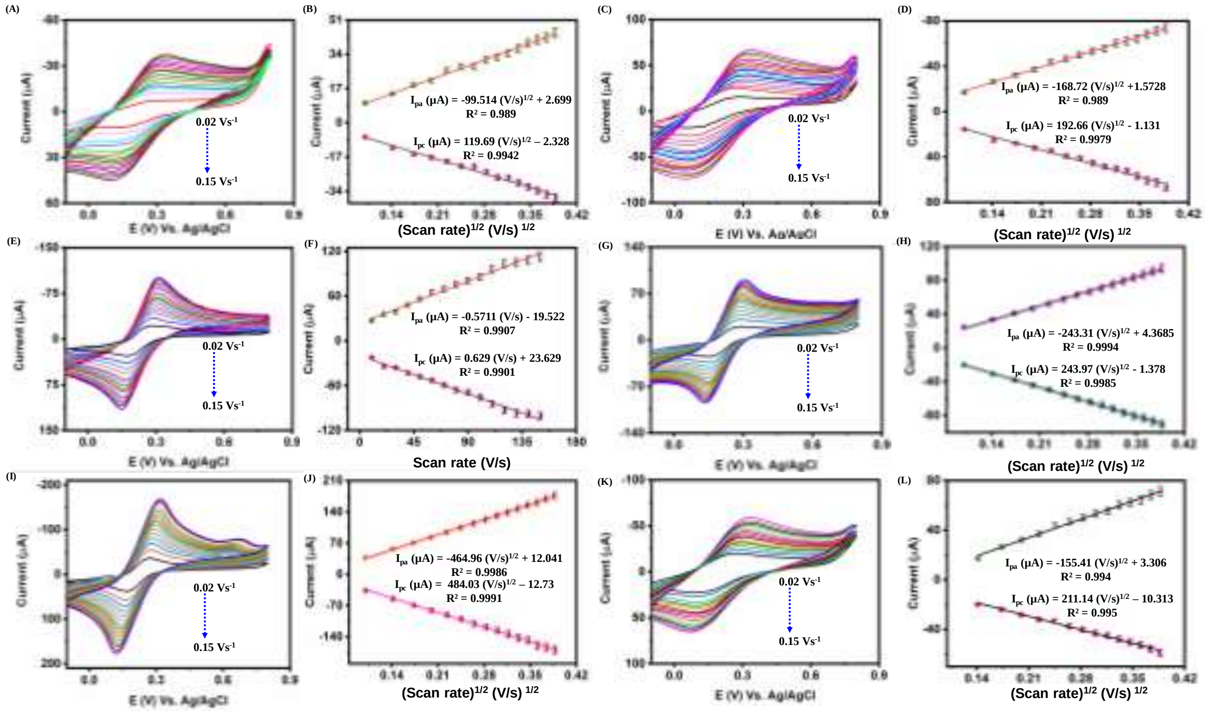

Fig. S2. Cyclic voltammetry curves for all the modified electrodes, **(A)** $AgBiS_2$ SPCE, **(B)** Corresponding correlation plot for the square root of scan rate (0.02-0.15 Vs^{-1}) versus peak current, **(C)** CNS SPCE, **(D)** Corresponding correlation plot for the square root of scan rate (0.02-0.15 Vs^{-1}) *versus* peak current, **(E)** $AgBiS_2$/CNS SPCE, **(F)** Corresponding correlation plot for the scan rate (0.02-0.15 Vs^{-1}) versus peak current, **(G)** $AgBiS_2$/CNS/GA/SPCE, **(H)** Corresponding correlation plot for the square root of scan rate (0.02-0.15 Vs^{-1}) versus peak current, **(I)** $AgBiS_2$/CNS/GA/Aptamer/SPCE, **(J)** Corresponding correlation plot for the square root of scan rate (0.02-0.15 Vs^{-1}) versus peak current, **(K)** Bare SPCE, and **(L)** Corresponding correlation plot for the square root of scan rate (0.02-0.15 Vs^{-1}) versus peak current. All the measurements were performed in the 0.1 M KCl $[Fe(CN)_6]^{3-/4-}$ (5 mM) system.

Conversion formula

$$\text{Concentration (g/L)} = \text{Concentration (M)} \times \text{Molecular weight (MW)}$$

$$\text{Concentration (g/L)} = \text{Concentration (g/L)} \times 10^{6}$$

Where:

- ❖ $1\ \mu M = 1 \times 10^{-6}$
- ❖ MW of CEA = 180,000 g/mol

Step 1: $C(M) = C\ (\mu M) \times 10^{-6}$

Step 2: $C(ng/mL) = C(\mu M) \times 10^{-6} \times 1.8 \times 10^{5} \times 10^{6} = C\ (\mu M) \times 180$

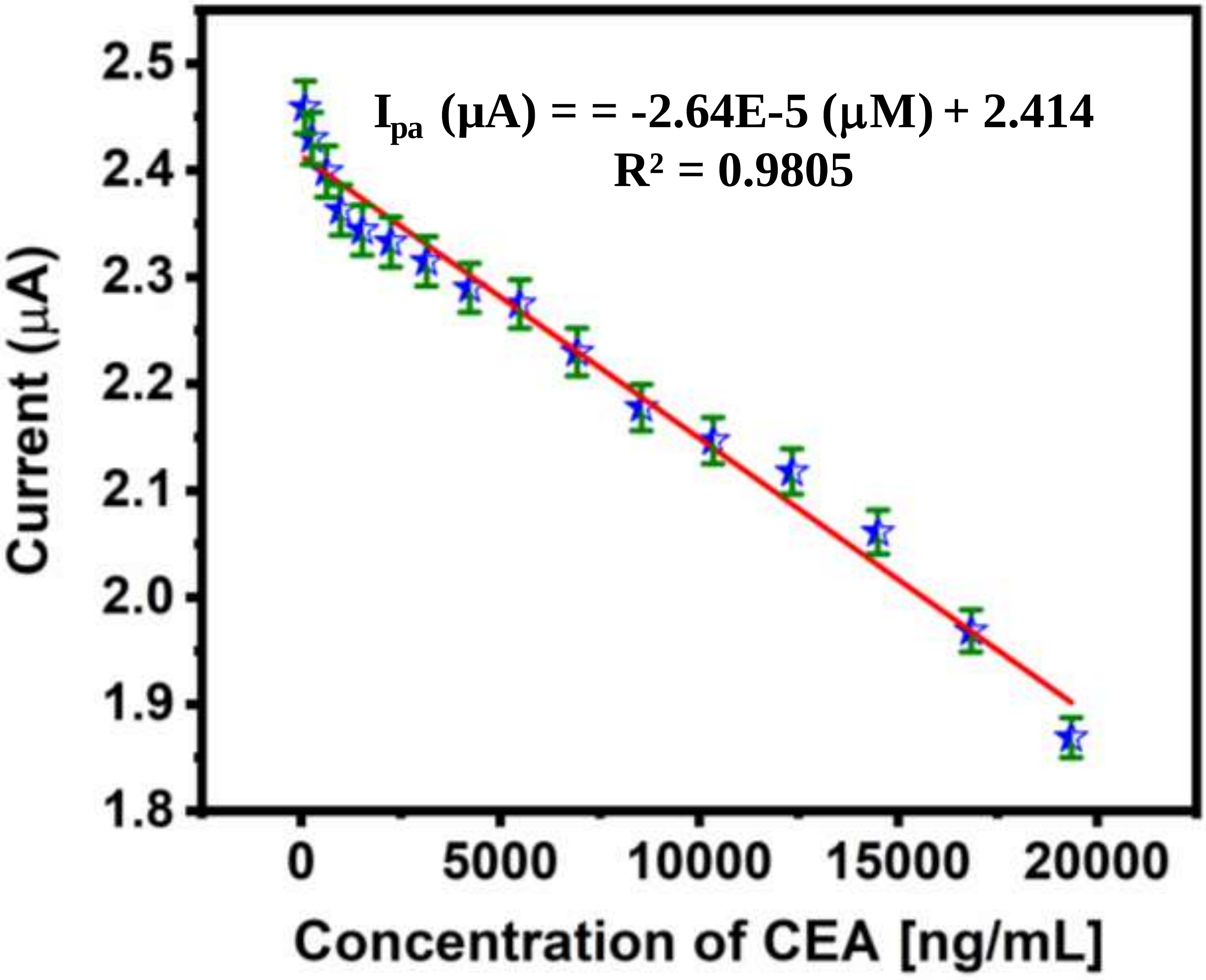

Fig.S3. Calibration plot for varying concentration of CEA [ng/mL] versus peak current.

Table S1. Recovery results for real sample analysis through aptamer-modified $AgBiS_2$/CNS/GA/Aptamer/SPCE electrode

**Sample**	**Added (mM)**	**Found (mM)**	**Recovery (%)**	**Relative standard deviation (RSD) (%)**
	0.05	0.0496	99.20	1.07 ± 3.02
**Blood serum**	0.15	0.1491	99.40	1.29 ± 2.17
	0.3	0.2942	98.06	2.18 ± 1.61
	0.45	0.4396	97.69	0.63 ± 1.58
	0.65	0.6480	99.69	3.69 ± 1.21

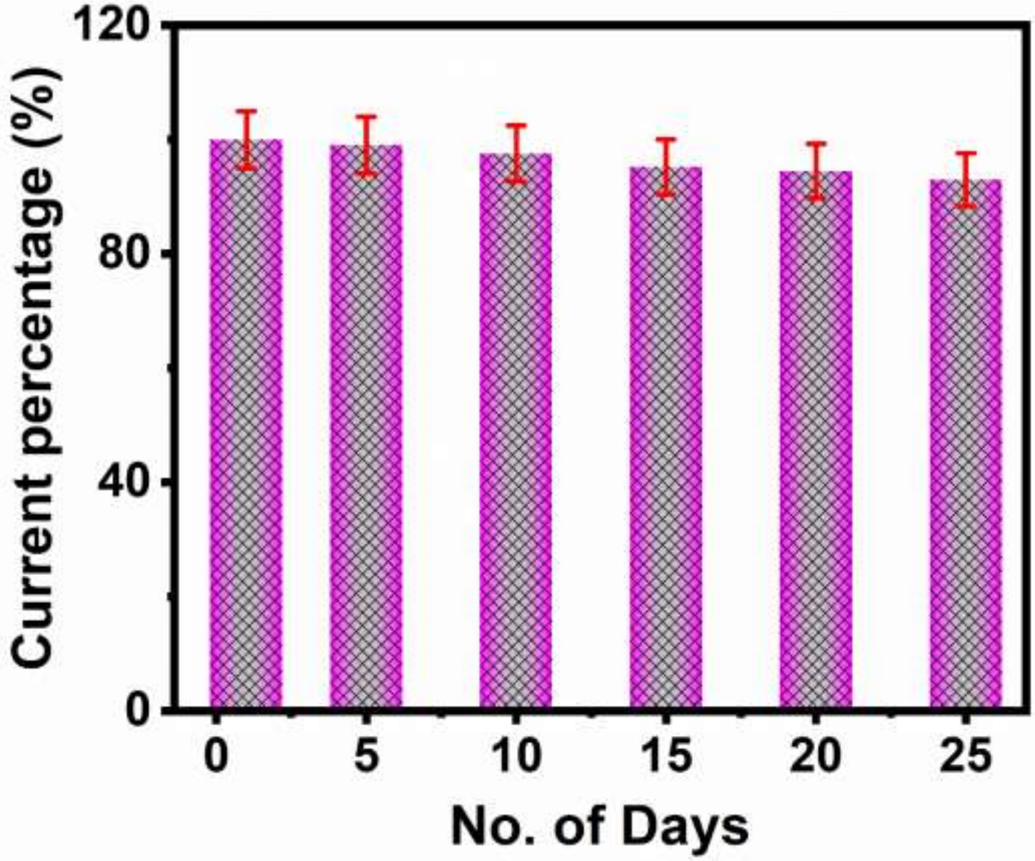

Fig. S4. Histogram for the storage stability analysis of the $AgBiS_2$/CNS/GA/Aptamer/SPCE were performed in the presence of 0.05 M PBS (pH-7.0) with 0.1 M KCl $[Fe(CN)6]^{3-/4-}$ (5 mM) system for 25 days.
